# Radon (^222^Rn) as tracer for submarine groundwater discharge investigation—limitations of the approach at shallow wind-exposed coastal settings

**DOI:** 10.1007/s10661-022-10462-5

**Published:** 2022-09-17

**Authors:** Michael Schubert, Jan Scholten, Matthias Kreuzburg, Eric Petermann, Mariele Lopes de Paiva, Dennis Köhler, Volker Liebetrau, John Rapaglia, Michael Schlüter

**Affiliations:** 1 Department Catchment Hydrology, Helmholtz Centre for Environmental Research GmbH - UFZ, Permoserstr. 15, Leipzig, 04318 Germany; 2 Coastal Geology and Sedimentology, Kiel University, Kiel, Germany; 3 Universidad Federal Do Rio Grande, Rio Grande, Brazil; 4 Alfred-Wegener Institute, Helmholtz Centre for Polar and Ocean Research, Bremen, Germany; 5 GEOMAR, Helmholtz Centre for Ocean Research, Kiel, Germany; 6 Department of Biology, Sacred Heart University, Fairfield, CT United States; 7 Federal Office for Radiation Protection (BfS), Berlin, Germany

**Keywords:** Submarine groundwater discharge, Radon, Tracer, Limitations, Wind speed and direction

## Abstract

Mapping radon (^222^Rn) distribution patterns in the coastal sea is a widely applied method for localizing and quantifying submarine groundwater discharge (SGD). While the literature reports a wide range of successful case studies, methodical problems that might occur in shallow wind-exposed coastal settings are generally neglected. This paper evaluates causes and effects that resulted in a failure of the radon approach at a distinct shallow wind-exposed location in the Baltic Sea. Based on a simple radon mass balance model, we discuss the effect of both wind speed and wind direction as causal for this failure. We show that at coastal settings, which are dominated by gentle submarine slopes and shallow waters, both parameters have severe impact on coastal radon distribution patterns, thus impeding their use for SGD investigation. In such cases, the radon approach needs necessarily to allow for the impact of wind speed and wind direction not only during but also prior to the field campaign.

## Introduction

Submarine groundwater discharge (SGD) occurs if the terrestrial hydraulic level is above the hydraulically connected sea level and a permeable coastal aquifer allows subsurface groundwater flow (fresh or brackish) to the sea. The global volume of SGD feeding into coastal oceans is estimated to be a factor 2–3 higher than the global river discharge to the sea (Kwon et al., 2014). Yet, even more important than the mere SGD volume is the solute transport associated with it. Several studies have shown that SGD-borne nutrient fluxes to the sea are in the same order as riverine nutrient input or even exceeding it (Knee & Paytan, 2011; Luo & Jiao, 2016; Rodellas et al., 2015; Santos et al., 2021; Seitzinger & Harrison, 2008). Hence, SGD can locally be the major driver for coastal primary bio-productivity (Rocha et al., 2015, 2016) and may even trigger the outbreak of harmful algal blooms (Hu et al., 2006; Lee et al., 2010; Luo & Jiao, 2016). A wide range of other pollutant fluxes (e.g., heavy metals, micronutrients, pesticides) can furthermore be associated with SGD (Black et al., 2009; Rahman et al., 2013; Trezzi et al., 2016). Consequently, localizing and quantifying SGD is of key relevance for the understanding of coastal environments and associated ecosystems.

The naturally occurring radionuclide radon (^222^Rn, t_½_ = 3.8 days) is widely used as tracer for SGD investigations (e.g. Burnett et al., 2006; IAEA-TECDOC-1595, 2008; Burnett et al., 2008; Stieglitz et al., 2010; Schubert et al., 2014; Petermann et al., 2018). Radon is continuously produced in any mineral matrix (thus, in any aquifer) by the decay of radium (^226^Ra). Since there is virtually no radon production in surface waters, radon concentrations in groundwater are generally about three orders of magnitude higher than in surface waters (including the coastal sea). That allows using elevated radon concentrations in the coastal sea for the localization of SGD spots and for the quantification of the local SGD rate.

While localizing SGD spots is based on only a qualitative assessment of the mapped radon distribution pattern, quantifying SGD rates requires setting up a radon mass balance for the coastal water volume located adjacent to the coastal section of interest. This mass balance (Eq. 1) must allow for all relevant radon sources and sinks (Burnett & Dulaiova, 2003). The related sources include the SGD-borne radon flux, i.e. the parameter of interest (F_SGD_; [Bq/m^2^/d]), diffusive radon flux from the sediments (F_Diff_; [Bq/m^2^/d]) and radon flux bound to river discharge (F_Riv_; [Bq/d]. The radon sinks include radon loss by radioactive decay (F_dec_; [Bq/d]), radon loss by atmospheric evasion (F_atm_; [Bq/m^2^/day]) and radon loss due to lateral and vertical mixing with sea water (in the following referred to as “offshore mixing”; F_mix_; [Bq/day]).

1 $${F}_{SGD}+ {F}_{Diff}+ {F}_{Riv}={{F}_{dec}+F}_{atm}+{F}_{mix}$$

*F*_*dec*_ is the best defined one among the six parameters. It can be derived from the radon inventory of the investigated water column and the radon decay constant. *F*_*Diff*_ can also be determined quite precisely based on batch experiments (Chanyotha et al., 2016) or on the radon concentration of bottom sediment pore water, i.e. the seawater/pore water concentration gradient (Cook et al., 2018). *F*_*Riv*_ is also quantifiable in a straightforward way as river water samples can in general be taken and river discharge rates can be measured easily.

In contrast to these three directly detectable parameters, *F*_*atm*_ and *F*_*mix*_ have to be derived indirectly based on proxy parameters. Consequently, they are the most uncertain parameters in the mass balance and uncertainties associated with the determination of the related radon fluxes results in potential errors (Rodellas et al., 2021). The study presented in this paper focussed on methodological challenges related to the parametrization of both atmospheric evasion and offshore mixing.

Generally, atmospheric evasion of radon from the coastal sea is driven by two factors, namely the air/seawater concentration gradient and the gas-specific radon transfer coefficient. The latter is (besides temperature and salinity; Schubert et al., 2012) a function of the extent of the air/water interface (i.e. the roughness of the water surface) and hence depending on the wind speed. Various model approaches exist that allow quantifying radon evasion from seawater (Bender et al., 2011; MacIntyre et al., 1995). However, comparison of the respective results shows that the estimated atmospheric losses vary by up to 58% depending on the model concept applied (Gilfedder et al., 2015). A common error source is that most conventional approaches do not account for storm events that occur prior to the actual radon mapping survey even though their aftermath may have significant impact on the mapped radon inventory (Petermann et al., 2018; Schubert et al., 2012, 2019). Thus, *F*_*atm*_ may introduce a substantial error in the radon mass balance, which then propagates to the finally calculated SGD (and matter) flux.

Quantifying radon loss by offshore mixing is error-prone, too. In coastal lagoons or estuaries, *F*_*mix*_ can be calculated based on the tidal prism, i.e. on the difference in the water volumes of lagoon or estuary between mean high tide and mean low tide (e.g. Schubert et al., 2015). However, this “tidal prism approach” is not applicable for open shorelines. In such settings, *F*_*mix*_ can be calculated from the minimum radon concentration at a fixed location in the coastal sea recorded during high tide. Still, this approach relies on the (potentially incorrect) assumption of maximum SGD during low tide and zero SGD during high tide (Burnett & Dulaiova, 2003).

Alternatively to the radon approach based on Eq. 1, SGD quantification is possible by physically capturing the discharging groundwater at the sea bottom by means of seepage meters (Lee, 1977; Taniguchi et al., 2003). However, in contrast to the radon approach, which results in data that allow integrating over time and space, seepage meter data result in SGD information restricted to point locations only (Cable et al., 2004; Povinec et al., 2012).

The activities discussed in this paper were carried out as a sub-study within the frame of a multidisciplinary long-term project that comprised numerous sampling campaigns executed by several groups between 2012 and 2018. This overall project aimed at localizing and quantifying SGD in the western Baltic Sea (Kreuzburg et al., submitted).

**Fig. 1:**
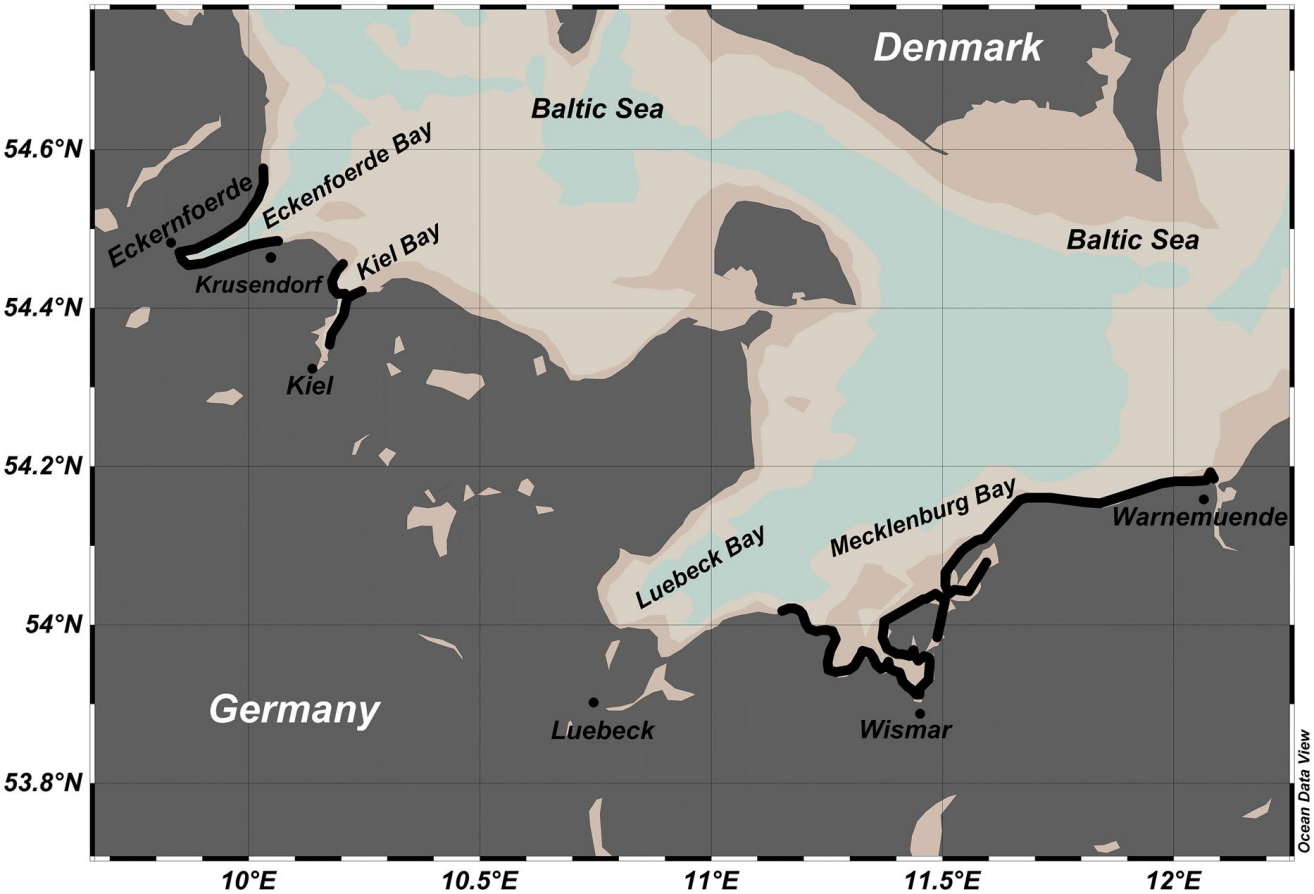
Location of the surveyed stretches of coastline (cruise tracks are marked by bold black lines) and the fixed sampling location at Krusendorf within the overall western Baltic setting

In our sub-study, we executed radon mapping surveys along three stretches of coastline within the larger project target area (April/May 2012 and April/May 2013) and recorded radon time series at a fixed coastal location (June 2015) (*cf*. Figure 1). Besides generating site-specific data, the main aim of our study was to evaluate the general applicability of the radon approach (*cf*. Equation 1) for SGD investigations in shallow wind-exposed coastal settings such as the western Baltic Sea. Focus of our field activities was on the Eckernförde Bay, an elongated basin in the western Baltic Sea.

## Methods

### Study area

The Baltic Sea is a humid marginal sea with an average depth of only about 50 m. Restricted water exchange to the North Sea and high terrestrial freshwater supply results in both an estuarine circulation and brackish seawater salinities. The sea is heavily impacted by human pressure as its overall catchment area is inhabited by more than 84 million people.

The larger study area, i.e. the western Baltic Sea, is characterized by year-around precipitation. Its morphology was formed during the late Weichsel ice advances leaving shallow coastlines dominated by end-moraines (Jensen et al., 2002). The water depths are below 25 m with the coastal slopes rising very gently towards the shoreline. Apart from a few major settlements (Warnemünde, Wismar, Kiel, Eckernförde), agriculture dominates the flat hinterland.

The tidal range in the western Baltic Sea is negligible (< 20 cm). Temporal sea level variations are predominantly controlled by wind intensity and direction. The wind has also major influence on coastal seawater flow paths and offshore mixing intensities. Shallow coastal settings are generally prone to coastal upwelling and downwelling caused by moderately to strongly blowing winds (Myrberg & Andrejev, 2003). Hence, wind-induced upwelling and downwelling are very common in the western Baltic Sea (Karstensen et al., 2014; Myrberg & Andrejev, 2003; Saderne et al., 2013). Specifically for the Eckernförde Bay, it was found that along-shore blowing winds cause downwelling cross-shore flow on one side of the bay and upwelling on the other side (Lehmann & Myrberg, 2008). Namely, in the north-western bay (Boknis Eck time-series station), upwelling was observed for wind directions between 190° and 260° at wind speeds between 4–6 m/s (Karstensen et al., 2014).

Although the main known transport routes of pollutants (input by rivers and the atmosphere) are well-monitored in the Baltic Sea (HELCOM, 2018), several studies indicate a considerable part of unmonitored water flows to the Baltic Sea, contributing to its eutrophication (Destouni et al., 2008; Hannerz & Destouni, 2006). One of these, to date, only poorly monitored water flows is SGD. A few SGD locations have been reported in the Baltic Sea, namely Laholm Bay (Sweden), the Gulf of Finland (Finland), Bay of Puck (Poland), Wismar Bay (Germany) and Eckernförde Bay (Germany) (Bussmann & Suess, 1998; Krall et al., 2017; Pempkowiak et al., 2010; von Ahn et al., 2021; Piekarek-Jankowska, 1996; Schlüter et al., 2004; Vanek & Lee, 1991; Kreuzburg et al., submitted). Specifically for the Eckernförde Bay, it was found that SGD originates from terrestrial freshwater aquifers extending offshore (Kreuzburg et al., submitted).

### Radon in seawater measurements

Mapping of radon distribution patterns in the coastal sea was done by (i) radon extraction from a continuous water pump stream applying a membrane extractor (MiniModule®, Membrana GmbH) (Schmidt et al., 2008) and (ii) radon measurement by means of a mobile radon-in-air monitor (RAD-7, Durridge). The setup had been proven suitable for the purpose during numerous field campaigns (e.g. Petermann et al., 2018). The radon-in-air concentrations detected on-site were converted into the associated radon-in-water concentrations by applying the temperature and salinity dependent radon partition correction (Schubert et al., 2012). In order to reduce uncertainties of radon results, two detection setups were run in parallel. Both monitors were set to a 15-min counting cycle.

Three stretches of coastline were investigated during the field activities. The coastline between Warnemünde and Mecklenburg Bay as well as the coastline of Kiel Bay were covered during a campaign lasting from April 25th to May 3rd 2012. The coastline of the Eckernförde Bay was covered during a campaign lasting from April 24th to May 1st 2013 (Fig. 1). In addition to these mapping surveys, radon time series were recorded at a fixed location in the Eckernförde Bay (Krusendorf) during three consecutive days (June 15th–17th 2015).

For coastal radon mapping, a boat was cruising close to the shoreline (distance < 100 m) with a speed of about 2 knots keeping the water depth between 1 and 3 m to avoid running aground. Seawater was continuously pumped from about 1 m water depth with a pumping rate of about 4 L/min. Besides continuous radon measurement, water salinity and water depth were constantly monitored using a CTD probe. The water temperature was recorded within the radon extraction module with temperature sensor (required for calculation of the water/air partitioning coefficient of radon).

For recording radon time series off Krusendorf, two RAD-7 s/MiniModule setups were placed in a rubber dinghy that was moored in 2 m distance from the mean beach water line (water depth about 1 m). Seawater was pumped continuously from 0.5 m water depth. Water salinity, water temperature, as well as wind speed were recorded simultaneously. Sea level data were obtained from the nearby Eckernförde gauge station (www.pegelonline.wsv.de).

### Radon in groundwater measurements

For the determination of the radon-in-groundwater endmember (required for F_SGD_ calculation in the radon mass balance; *cf*. Equation 1), eight groundwater samples were taken from beaches in the Eckernförde Bay. The water was sampled from a depth of about 50 cm using push point piezometers. All samples were analysed for radon by means of liquid scintillation counting following the procedure described in Purkl and Eisenhauer (2004).

### Complementary data collection

Direct measurements of SGD rates and measurements of submarine pore water compositions were carried out in the Eckernförde Bay within the frame of the long-term project that our sub-project was part of. Both the methodical approaches and the related results are discussed in detail in Kreuzburg et al. (submitted). For the sake of completeness, only a few related facts shall be recapped here.

For the physical determination of SGD fluxes, Kreuzburg et al. (submitted) deployed seepage meters at four locations in the Eckernförde Bay, namely at Hemmelmark, Langholz, Kiekut and Krusendorf (*cf*. Figure 3). At Krusendorf, the seepage meters were deployed around the location of the rubber dinghy installed for our radon time series recording (*cf*. Section 2.2). Furthermore, Kreuzburg et al. (submitted) took 23 pore water samples from the marine bottom sediments (sediment depth 20–30 cm) in the Eckernförde Bay close to the beachline. Salinity, conductivity and temperature of the samples were determined directly on site by means of a hand-salinomter (WTW COND 3310).

## Results

### Radon in seawater

**Fig. 2:**
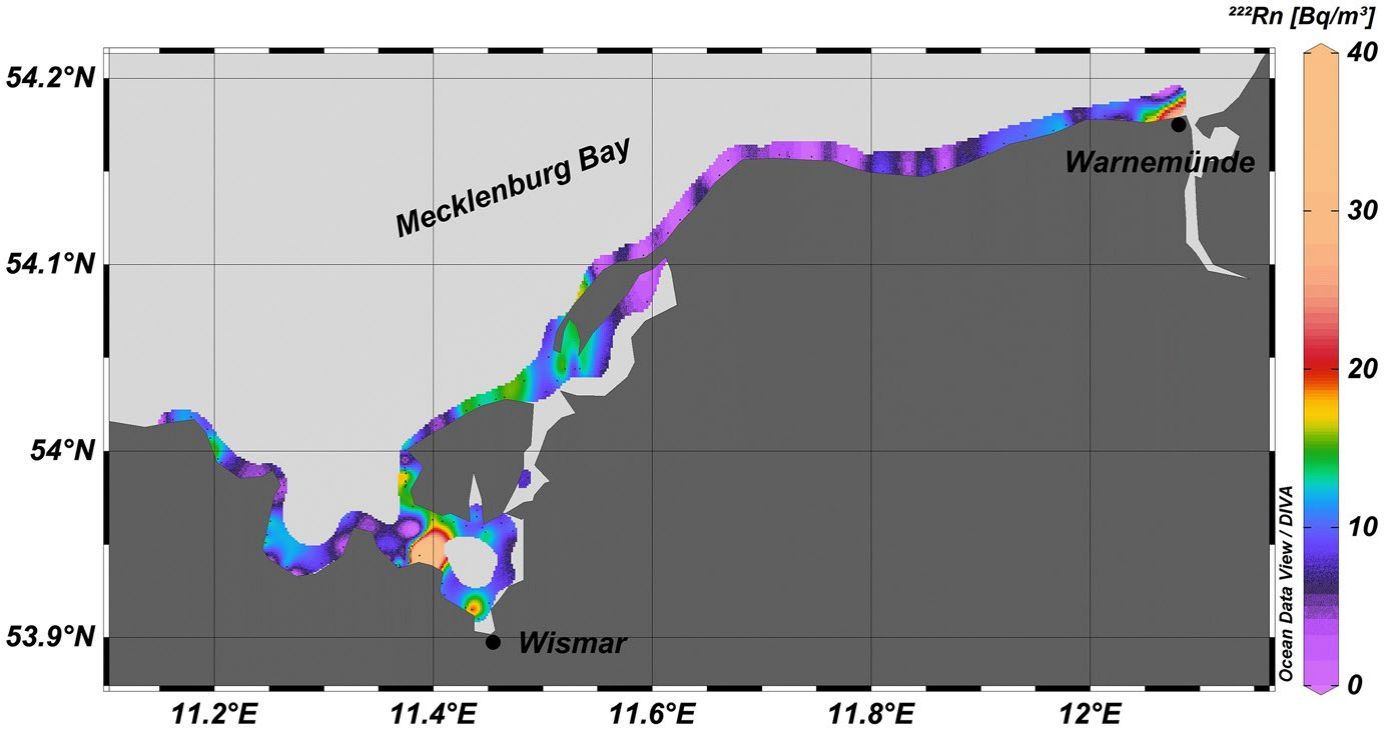
Radon distribution pattern in coastal surface water mapped in the Mecklenburg Bay. Elevated radon concentrations were observed off Wismar and Warnemünde

**Fig. 3:**
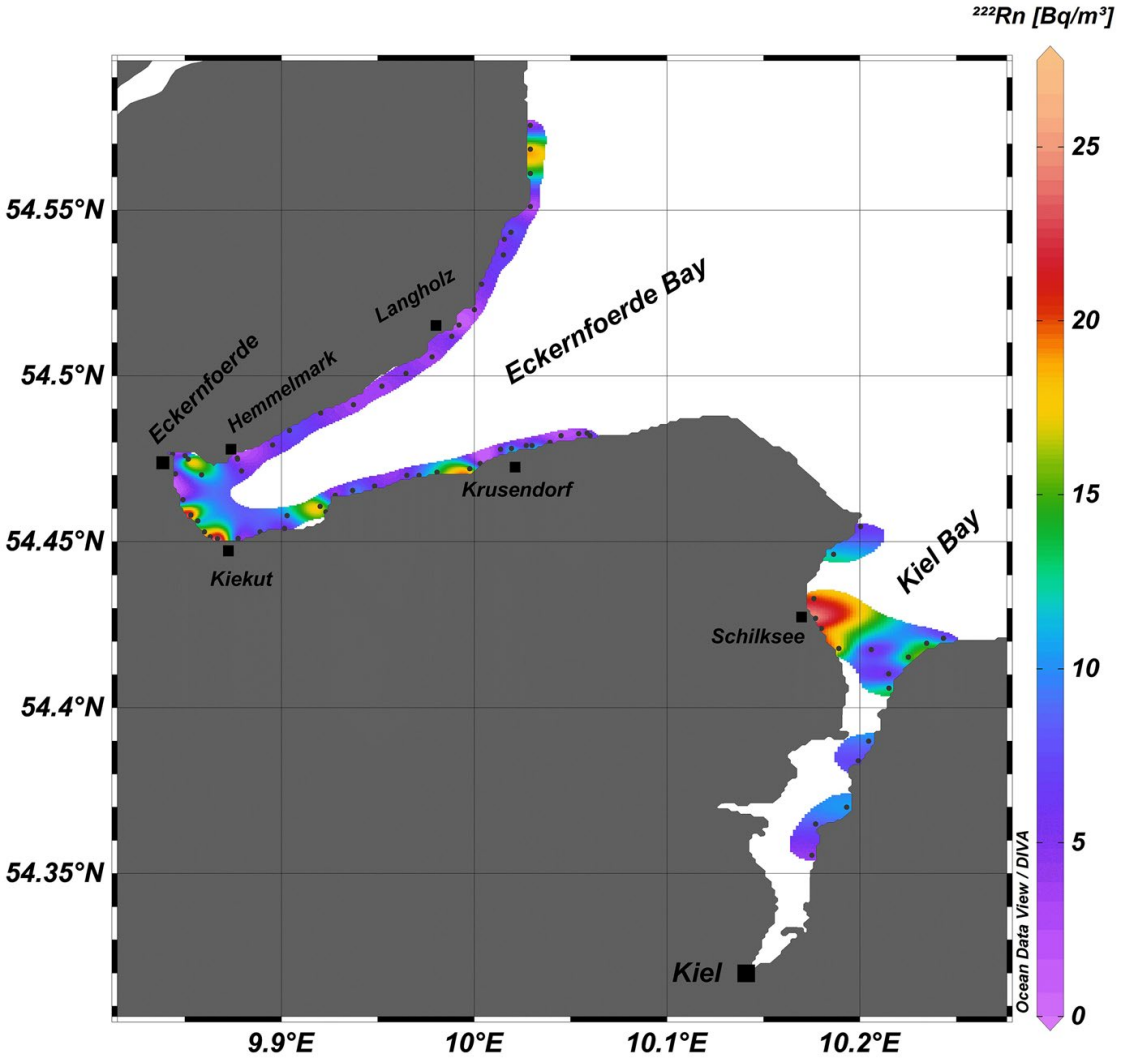
Radon distribution patterns in coastal surface water mapped in the Bay of Kiel and the Eckernförde Bay. Elevated radon concentrations were observed off Schilksee (Bay of Kiel) and Kiekut (Eckernförde Bay)

Covering three stretches of coastline (*cf*. Figure 1), the radon mapping surveys revealed seawater radon concentrations ranging from the natural offshore background (i.e. radon that is only supported by decay of ^226^Ra dissolved in the seawater) of about 5 Bq/m^3^ to peak concentrations around 65 Bq/m^3^ with an overall mean of 9.1 ± 6.4 Bq/m^3^. Based on a coastal radon background value defined as the detected overall mean plus one standard deviation (i.e. 15.5 Bq/m^3^), elevated radon concentration were located off Warnemünde, off Wismar, at the western shoreline of the Bay of Kiel near Schilksee and in the southwestern Eckernförde Bay near Kiekut (Figs. 2 and 3). (At Schilksee, this indication is in accordance with visible groundwater discharge on the beach: During periods of low sea level, groundwater can be seen seeping out of the beach face.)

**Fig. 4:**
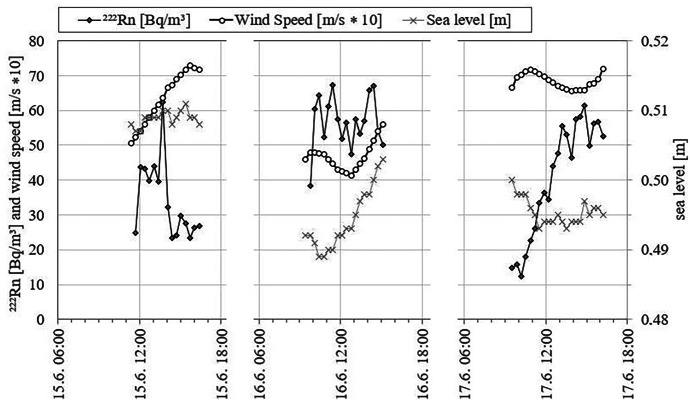
Radon concentrations in coastal surface water, wind speed and sea level at the fixed location off Krusendorf (note that for the sake of scaling wind speed was multiplied by 10)

The values of the radon time series recorded at the fixed location off Krusendorf were found to be in the same range as the mapping results, however, with a higher mean of 42.5 ± 17 Bq/m^3^ (Fig. 4).

**Fig. 5:**
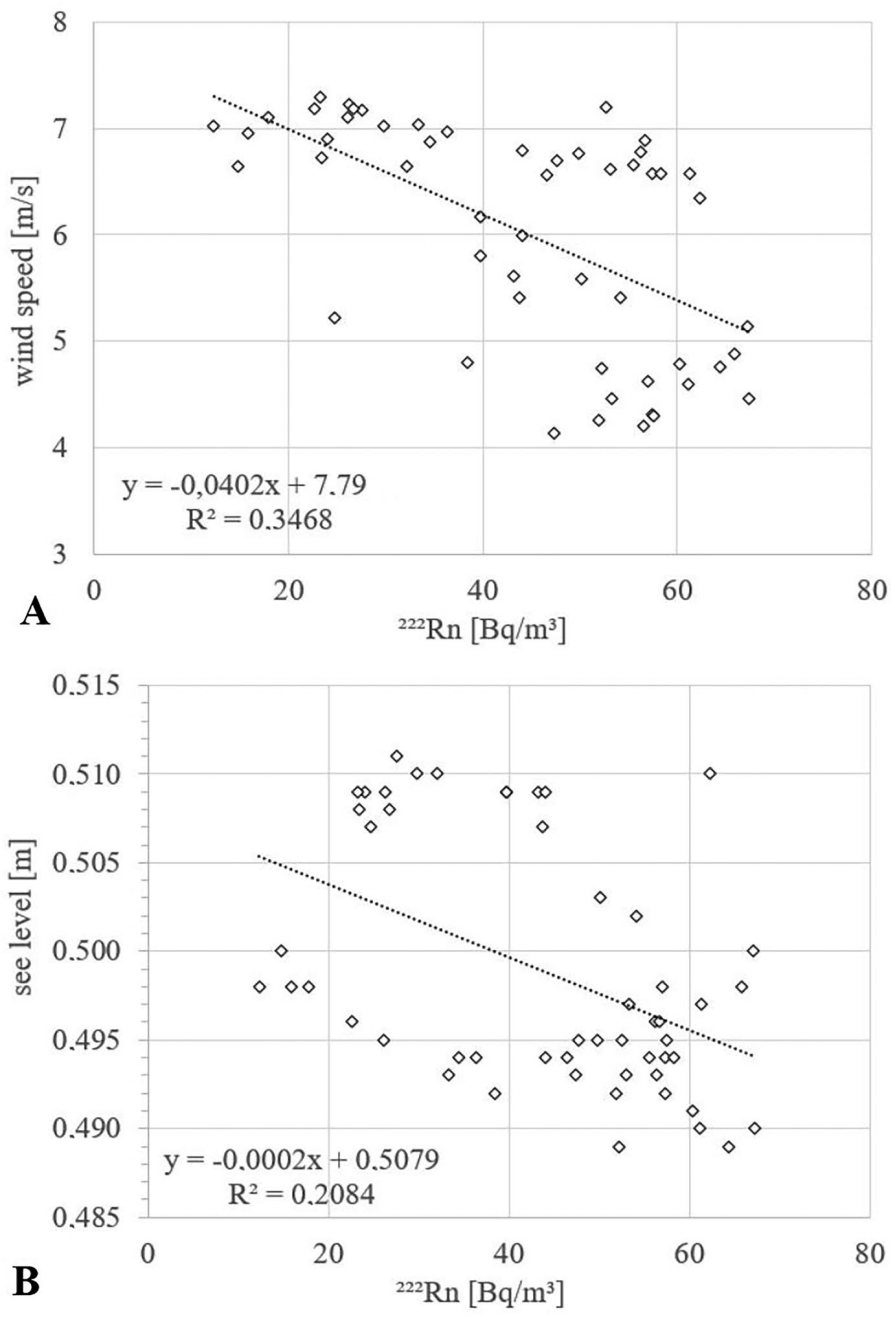
Relations between radon in seawater and **A** wind speed and **B** sea level, respectively, at the fixed location off Krusendorf

During recording of the Krusendorf time series, the local wind speed varied between about 4 and 7 m/s. Plotting the datasets radon concentration vs. wind speed reveals a negative but rather poor correlation of the two parameters (R^2^ = 0.35; Fig. 5A).

As the tidal range is almost negligible in the western Baltic Sea, cyclic tidal pumping was not expected to be influential for the SGD rate. Evaluating radon concentration vs. sea level revealed consequently an only poor (negative) correlation as well (R^2^ = 0.21; Fig. 5B). This is in contrast to most other coastal radon time series studies, which report the radon concentration to vary inversely with the tides due to tidal pumping (Burnett & Dulaiova, 2003; Dulaiova et al., 2008; Rocha et al., 2015).

### Radon in groundwater

Analysis of the eight groundwater samples taken along Eckernförde Bay beaches revealed radon endmember concentrations with a considerable variance. The following concentrations were found: at Hemmelmark 11.2 ± 2.0 kBq/m^3^ (*n* = 2), at Langholz 13.1 ± 2.1 kBq/m^3^ (*n* = 4) and at Krusendorf 0.9 ± 0.28 kBq/m^3^ (*n* = 2). A study that had been executed previously in Eckernförde Bay (Purkel & Eisenhauer, 2004) had revealed comparable radon in groundwater concentrations, however in a smaller range (5.9–6.9 kBq/m^3^).

### Complementary data

For the sake of clarity and completeness, we briefly recap here the seepage meter results and the salinity of submarine pore water data, which are discussed in detail elsewhere (Kreuzburg et al., submitted).

**Fig. 6:**
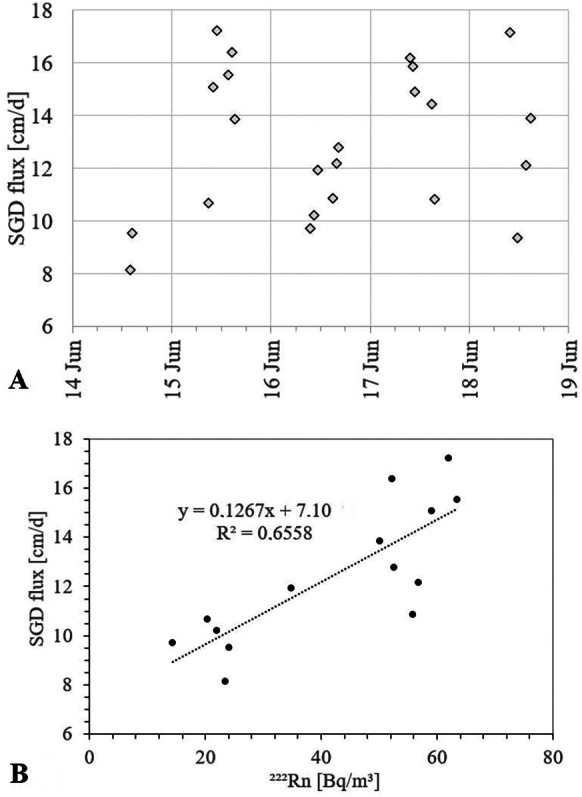
**A** SGD seepage meter fluxes and **B** relation between SGD seepage meter fluxes and radon time series data off Krusendorf

The seepage meter measurements that were carried out between 2013 and 2018 at four sites in the Eckernförde Bay resulted in SGD fluxes covering a wide range, namely at Hemmelmark 18.8 ± 16 cm/day (range 0.5–80 cm/day), at Langholz 33.3 ± 42.7 cm/day (range 1.6–173 cm/day), at Kiekut 21.6 ± 22 cm/day (range 0.8–128 cm/day) and at Krusendorf 11.6 ± 5.3 cm/day (range 2.6–29.3 cm/day). The seepage meter data recorded at Krusendorf specifically during our radon time series measurement (June 15th–17th 2015) ranged between 8.2 and 17.2 cm/day with a seemingly cyclic behaviour (Fig. 6A). Plotting the seepage meter dataset vs. our recorded radon time series data revealed a positive correlation (R^2^ = 0.66; Fig. 6B).

**Fig. 7:**
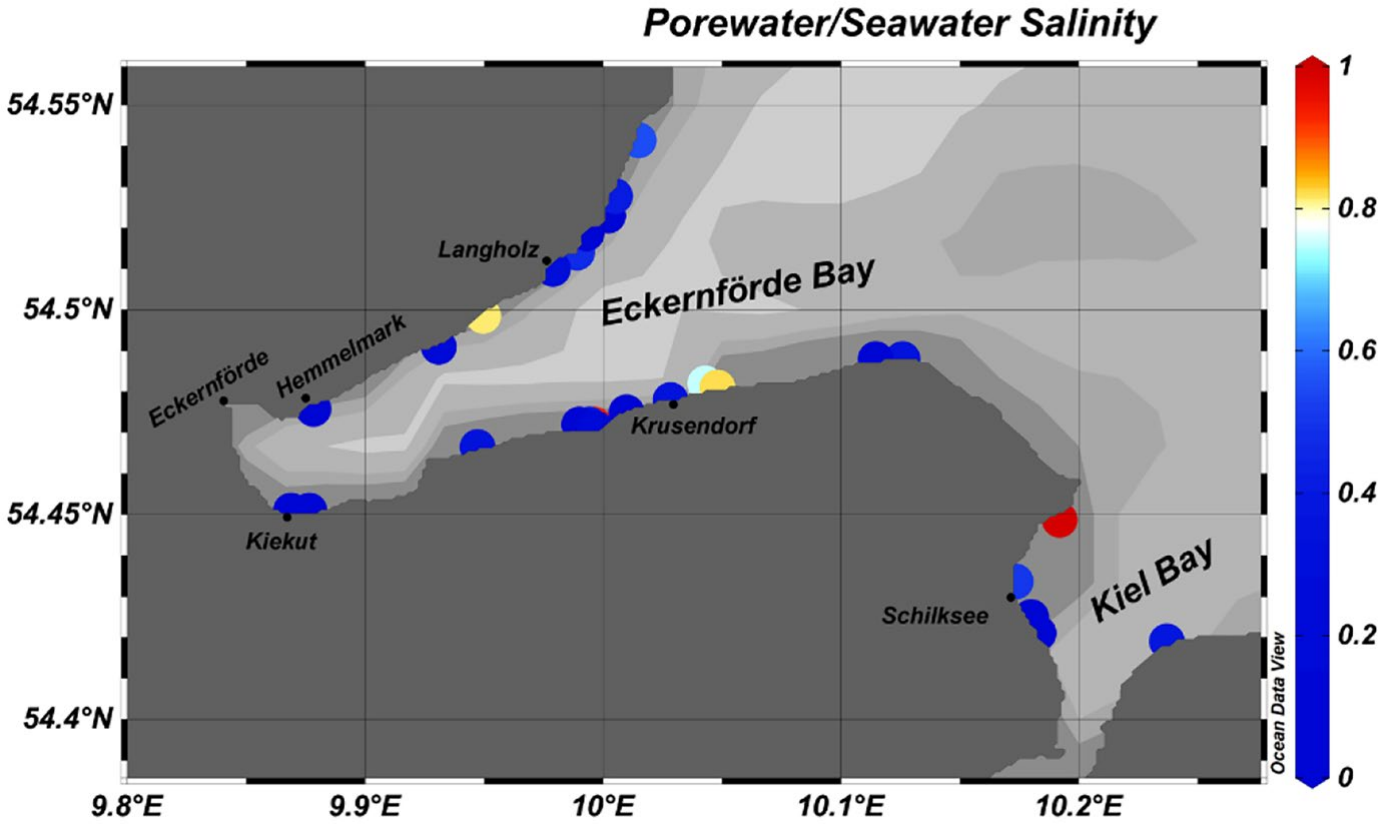
Ratio of sediment pore water salinity and ambient seawater salinity (modified after Kreuzburg et al., submitted)

The submarine pore water quality measurements revealed that at 19 out of the investigated 23 locations the pore water showed significantly lower salinities than the seawater sitting on top of the sediments (Fig. 7). This confirms a significant share of fresh groundwater in the sediment pore water suggesting the occurrence of SGD.

## Discussion

### Coastal radon distribution patterns

As mentioned above, our study aimed mainly at evaluating the general applicability of the radon approach for SGD investigation in shallow wind-exposed coastal settings such as the western Baltic Sea. Thus, our discussion focuses on the area from which the most related data were available, i.e. the Eckernförde Bay.

Based on the conceptual model assumptions of the radon approach, we expected to find positive radon anomalies at locations were low-saline sediment pore water and elevated seepage meter fluxes had been found by Kreuzburg et al. (submitted). This expectation was not met by the data, though.

Generally, the radon concentrations mapped along the coastline of the Eckernförde Bay (in particular at its north-western coast) were at background level, thus, indicating the absence of SGD (*cf*. Figure 3). At the same time, the notably low pore water salinities found there do indicate freshwater discharge (*cf*. Figure 7). Isolated elevated ^222^Rn concentrations were recorded near Kiekut. However, they are only based on single data points. While indicating qualitatively the presence SGD, the data would not allow an unequivocal quantitative assessment of SGD rates. At the same time, seepage meter measurements near Kiekut had revealed a SGD rate of 11.3 ± 5.3 cm/day. An additional radon survey, carried out under low-wind conditions, might confirm these seepage meter data. The contradiction between the results of radon mapping and seepage meter measurements is even more noticeably near Langholz, where, on the one hand, the seepage meter results revealed high discharge rates of 33.3 ± 42.7 cm/day but where, on the other hand, no positive radon anomaly was detected.

Commonly, the lack of positive radon anomalies close to spots of (physically) confirmed SGD occurrence is explained with the inter-annual variability of SGD flux rates. Reduced SGD can result from periods of extensive freshwater abstraction or drought, i.e. from conditions that moderate the hydraulic gradient between terrestrial groundwater and seawater (Rocha et al., 2015; Santos et al., 2009). However, freshwater in the area of the Eckernförde Bay is normally abstracted from deep confined Tertiary aquifers that have limited connection to surficial aquifer domains (Jensen et al., 2002). Furthermore, in the years of our field studies, no drought occurred and annual precipitation varied in a relatively narrow range (historic data for Kiel, ca. 20 km NE of the Eckernförde Bay show for 2012 716 L/m^2^, for 2013 664 L/m^2^, for 2014 782 L/m^2^, for 2015 866 L/m^2^, for 2016 716 L/m^2^ and for 2017 888 L/m^2^; http://www.wetterkontor.de). This rather steady situation is confirmed by the seepage meter measurements executed at Hemmelmark in 2013, 2014, 2016 and 2018, which revealed similar SGD fluxes (Kreuzburg et al., submitted), thus suggesting a rather constant SGD flux on an inter-annual time scale. Hence, others processes, which are discussed in the following, must be responsible for the fact that the mapped radon distribution pattern failed to indicate locations with proven occurrence of SGD within the Eckernförde Bay.

Applying the conceptual mass balance model, given in Eq. 1, we can in our particular case assume F_Riv_ and F_Diff_ to be negligible. There are no major rivers discharging to the Eckernförde Bay and the coastal sediments consist of glacial tills and sands generally low in ^226^Ra and with little compositional variations. Thus, Eq. 1 simplifies in the given case to Eq. 2.

2 $${F}_{SGD}={F}_{atm}+{F}_{dec}+{F}_{\mathrm{mix}}$$

Equation 2 suggests that low radon concentrations in spite of proven groundwater seepage are a result of radon losses from the coastal seawater that were not accounted for in the radon mass balance. With radon decay (*F*_*dec*_) being quantitatively defined, the two remaining radon sinks atmospheric evasion (*F*_*atm*_) and offshore mixing (*F*_mix_; defined as the inverse of the water residence time) were looked at more closely.

### Radon loss by atmospheric evasion

**Fig. 8:**
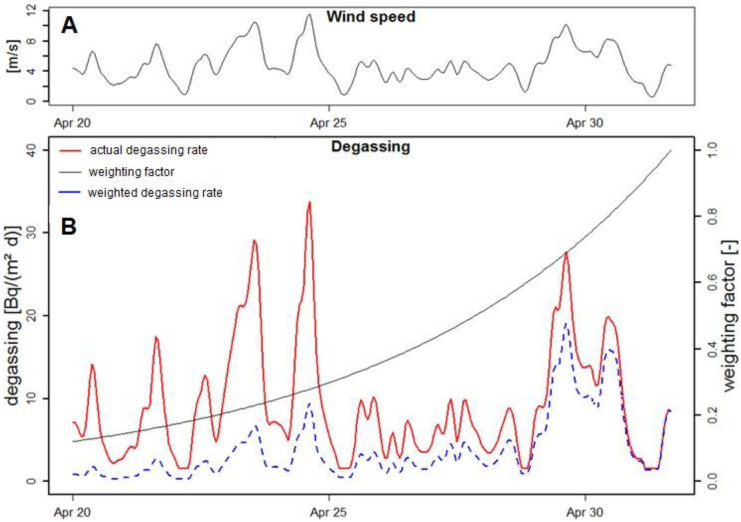
**A** Wind speed in the Eckernförde Bay between April 20th and May 1st 2013. **B** Radon degassing (red line) derived from the wind speed data. The factor for weighting the impact of previous degassing (black line) shows an exponential decrease with increasing temporal distance to the moment of sampling (May 1st at 04:00 p.m.) at which the weighting factor approaches 1. The weighted degassing (blue dashed line) represents the product of degassing and weighting factor

Radon loss by atmospheric evasion is mainly controlled by the wind speed with higher loss rates during times of strong winds and vice versa (MacIntyre et al., 1995). Even though a range of approaches to estimate atmospheric evasion exist, the uncertainties associated with the resultant radon fluxes lead to potential errors (Rodellas et al., 2021). Evaluating the wind speed data for the Eckernförde Bay reveals wind speeds ranging between 1 m/s and 11 m/s (i.e. peak values of Beaufort force 6, “strong breeze”) both during our radon survey (which ended May 1st 2013) and in the week before it (Fig. 8A). This implies that strong winds caused substantial radon evasion with sustaining impact on the seawater radon concentration already prior to our survey.

We want to exemplify this “memory effect” by looking closely at the distinct data point, May 1st at 04:00 p.m. (^222^Rn = 17 Bq/m^3^; ^222^Rn inventory = 34 Bq/m^2^; Fig. 8B). By including not only the wind speed recorded at this specific point in time, but by also considering the wind speed data recorded in the previous days, we were able to calculate an effective radon-degassing rate considering all degassing that had happened prior to sampling. In fact, we considered the impact of degassing related to all wind events that had occurred during the 10 days prior to our sampling campaign. The impact of each individual event was weighted by a factor. The weighting factor was parameterized as described in detail by Petermann et al. (2018) and Schubert et al. (2014). As shown in Fig. 8B, the wind events becomes less relevant for a measured radon concentration the longer ago they occurred before sampling. In our example, the intense degassing, which occurred between April 29th and April 30th, reduced the radon concentration in the seawater measured on May 1st at 04:00 p.m. significantly, even though the wind speed (and hence the degassing rate) at the actual moment of sampling was rather low.

Consequently, it has to be assumed that the radon concentrations detected during the survey in the north-western part of the Eckernförde Bay (i.e. at Langholz) were strongly affected by wind-induced degassing that had occurred prior to the actual sampling. Hence, if radon data evaluation is ignoring this prior degassing, the mapped seawater radon pattern does not indicate the verifiably present SGD location.

### Radon loss by offshore mixing

In “Study area” section, we discussed wind-induced offshore mixing as it is typical for shallow coastal settings. Such offshore mixing has to be considered a highly influential process along the three stretches of coastline investigated in our study, in particular in the Eckernförde Bay. A recent study (Dietze & Löptien, 2021), which investigated offshore mixing in the Eckernförde Bay between 2000 and 2018 in high-resolution, showed highly variable water residence times (i.e. the time since the water entered the bay) and water ages (i.e. the time since the water was last in contact with the atmosphere). The authors identified the wind direction as key driver for the intensity of variation. For instance, the abovementioned strong (north-easterly) winds that occurred in the week preceding our radon survey in the bay caused the water residence time in the inner bay to drop to under 10 days (Dietzen & Löptien, 2020). Wind-induced offshore mixing caused the discharged groundwater (enriched in radon) to be rapidly mixed with bay waters low in radon, resulting in less pronounced (or completely levelled-out) radon concentration anomalies close to the (physically identified) coastal SGD spots.

In our given case, a quantitative relation between wind-induced offshore mixing and coastal radon concentrations is difficult to estimate based on the available data. Nevertheless, as an approximation, we calculated the mixing effect on the coastal radon concentrations for three conceptual water mixing rates. For convenience, the radon inventory of the coastal water volume (*RnI*) was calculated assuming a negligible radon degassing rate (*F*_*atm*_ = 0 Bq/m^2^d) and a constant SGD flux (*F*_*SGD*_ = const.). Based on Eq. 3, we estimated the SGD-induced radon flux into the coastal water volume. The radon mixing loss (*F*_*mix*_) was then calculated for three different coastal water residence times $\tau$ (1, 5 and 100 days). The offshore mixing rate was defined as the inverse of the coastal water residence time. We solved Eq. 3 inversely going back in time with steps of *t* = 1 day (Eq. 3). This approach takes account of the dependency of *RnI*_*t*_ on radon decay (*F*_*dec*_), radon degassing (*F*_*atm*_) and offshore mixing (*F*_*mix*_). The radon inventory is defined as the value towards which *RnI* converges as it reaches a steady state, i.e. for *t* → -∞. Finally, the resulting radon inventories were converted to radon concentrations by dividing them by the water depth.

3 $$Rn{I}_{t-1}={F}_{de{c}_{t}}+{{F}_{{atm}_{t}}+{F}_{mix}}_{t}+{F}_{SGD}$$

**Fig. 9:**
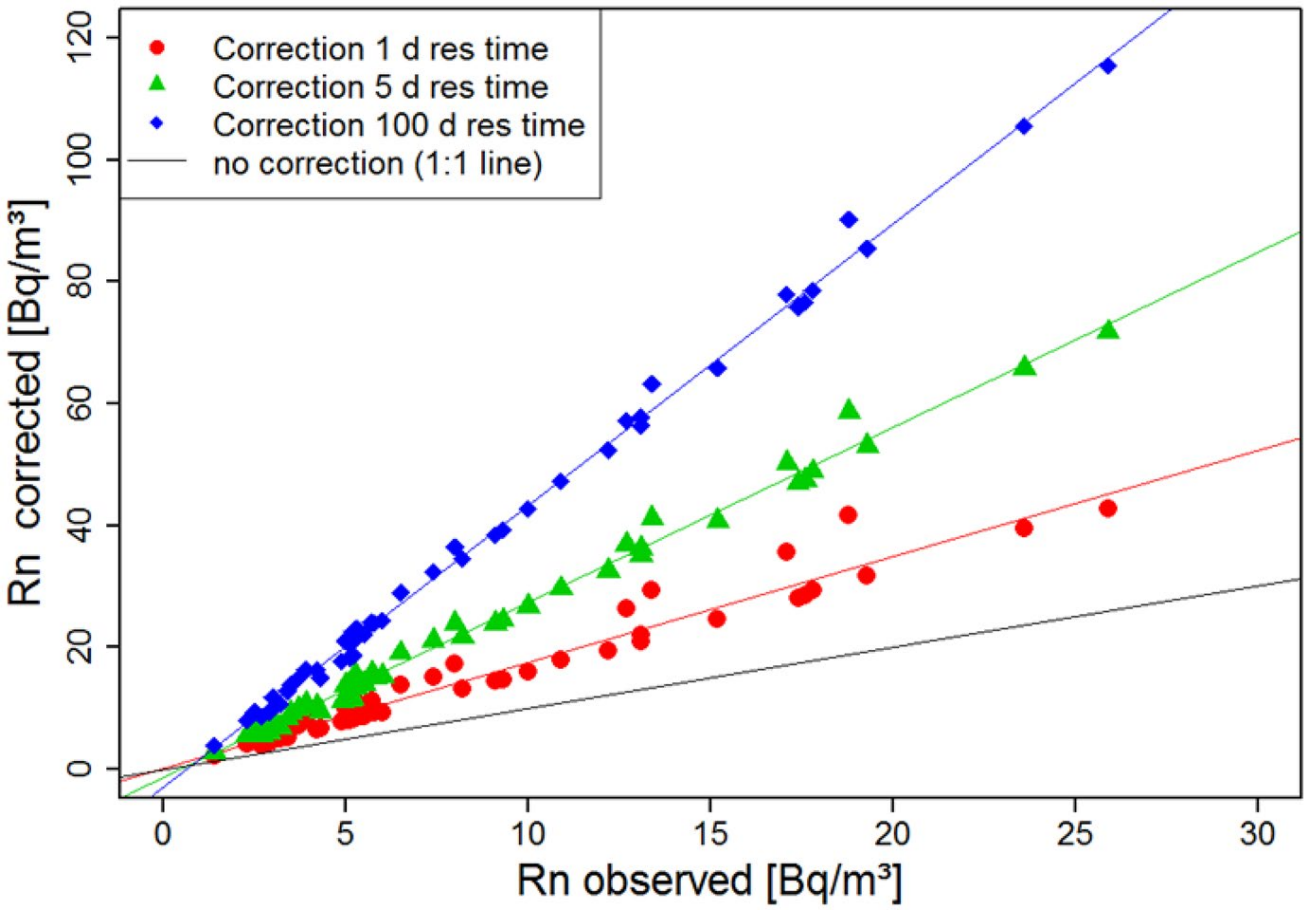
Observed (black line) and corrected radon in seawater concentrations allowing for loss by offshore mixing and radon decay (assuming *F*_*atm*_ = 0 Bq/m^2^ day and a constant SGD flux rate). Three different water residence time scenarios (1, 5 and 100 days) were considered

For all the three residence time scenarios, the corrected radon concentrations are significantly higher than the observed ones (Fig. 9). The corrected concentrations increase on average by a factor of 1.8 for $\tau =1 \mathrm{day}$, 2.6 for $\tau =5\ \mathrm{days}$ and 4.1 for $\tau =100\ \mathrm{days}$. Longer residence times are equivalent to lower mixing losses and do hence support SGD-induced positive anomalies in the coastal radon concentration distribution. At the same time, they result in higher decay losses.

The relative variability of the corrected radon concentrations with respect to linear regression (as displayed in Fig. 9) is a result of varying effective degassing rates. Samples that were predominantly exposed to higher wind speeds during the previous 10 days are attributed with higher degassing corrections (thus plotting above the regression line) than samples that were mainly exposed to lower wind speeds (thus plotting below the regression line).

The three conceptually calculated scenarios show that the radon approach (*cf*. Equation 1) is very sensitive to the mixing time scales of the coastal water. Quantification of the water mixing is difficult as changes in the mixing regime can occur fast. This is demonstrated in the following based on the time-series data recorded at a fixed location off Krusendorf.

At the time-series station, seawater radon concentrations varied by a factor of about 5.5. Due to the lack of tidal influence, no tide-related cyclical change in radon concentration was observed (*cf*. Figures 4 and 5B). Furthermore, the data reveal only a minor (negative) correlation between radon and wind speed (*cf*. Figure 5A). As the wind speed was relatively constant during the 3 days of recording (mean = 6 ± 1.1 m/s), the radon atmospheric evasion at this fixed location was presumably relatively constant. However, on June 17th 2015, the wind speed remained nearly constant at 6.8 m/s but the radon concentration changed by a factor of about 4.5 (ranging between 14 and 61 Bq/m^3^) (*cf*. Figure 4). On the other hand, on June 16th 2015, again a day with rather constant wind speed (ca. 6.8 m/s), the radon concentration varied only slightly around 55 Bq/m^3^. Thus, the observed changes in seawater radon concentration are most probably neither related to seawater level changes nor to changes in wind speed.

As illustrated in Fig. 6B, the Krusendorf time-series revealed a positive *f*(t) correlation between detected seawater radon concentration and physical SGD flux as determined by seepage meters (R^2^ = 0.67). Such relation is reasonable, as a higher SGD rate causes more SGD-borne radon to be supplied to the coastal water. At the same time, the plot displayed in Fig. 6B implies some questionable inferences: Extrapolating the best-fit regression line of the SGD/radon relation would predict negligible radon concentrations at a SGD flux rate of about 7 cm/day, which is implausible. We would rather expect the best-fit line to intersect (at a SGD flux = 0 cm/d) with the x-axis at a value ^222^Rn > 0 Bq/m^3^ due to the presence of radon that is not attributable to SGD but to diffusive radon input, i.e. the intercept with the y-axis should be negative. Furthermore, Fig. 6B reveals quite some variability in the SGD/radon correlation: We observe a wide range of radon concentrations for about the same SGD flux rate. For instance, for a SGD flux of about 10 cm/day, we detected radon concentrations between about 15 and 60 Bq/m^3^; a SGD flux of about 12 cm/day was associated to radon concentrations between 35 and 55 Bq/m.

The recorded huge spread of radon concentrations (at an assumed steady SGD flux), on the one hand, and the implausibility of radon variance due to wind speed and sea level changes, on the other hand, suggest offshore mixing as most likely process governing the seawater radon concentration at Krusendorf. On June 16th 2015, when radon concentrations remained relatively constant at around 55 Bq/m^3^, the wind was blowing with a rather constant speed from a north-westerly direction, i.e. approximately perpendicular to the coastline. In the early morning of June 17th 2015, the wind picked up a bit but, more importantly, changed direction temporarily to south-west, i.e. parallel to the shoreline. This shore-parallel wind probably caused temporal downwelling of surface waters resulting in rapid removal of freshly discharged groundwater (rich in radon) by offshore seawater (low in radon). Thus, the coastal radon concentration dropped significantly in the early hours of the day and the radon time series started (at 09:00 a.m.) with values of only around 15 Bq/m^3^.

## Conclusions

It was found that in shallow wind-exposed coastal settings, radon might not be applicable as SGD tracer, since wind speed and wind direction have a non-quantifiable impact on both atmospheric evasion and offshore mixing. In our study, the radon approach failed to identify several locations of known SGD occurrence in the Eckernförde Bay, Baltic Sea. We show that strong wind events occurring several days prior to a radon survey may have a strong sustaining impact on the radon distribution pattern mapped during the survey. Therefore, it can generally be stated that radon surveys, which are conducted a few days after storm events, may fail to identify SGD locations due to the still noticeable radon loss by degassing. This highlights the need to account for any potential radon degassing prior to a radon survey.

We also observed that wind-induced offshore mixing of coastal waters may significantly hamper the build-up of SGD-borne radon concentration anomalies in coastal seawater. In particular in shallow coastal settings, wind-induced water circulation (such as upwelling or downwelling) prompts offshore mixing so that radon-enriched coastal waters get mixed with offshore waters low in radon, thus complicating the identification of SGD locations based on positive radon anomalies. Besides the wind speed (during and prior to the survey), the wind direction has to be taken into consideration here.

Based on our detailed results from the Eckernförde Bay, we assume that our preliminary radon surveys in Kiel Bay and Mecklenburg Bay failed to detect SGD locations for the same reasons. Since the radon approach does not seem to be fully applicable in shallow and wind-exposed coastal settings, other techniques (such as geo-electrical tomography) are required to obtain a more complete picture on the extent of SGD occurrences in settings such as the south-western Baltic Sea.

Our study focused on limitations of the radon approach in shallow wind-exposed coastal settings due to offshore mixing and atmospheric evasion. In the case of radon surveys executed in lakes and slow flowing rivers, the situation is different. Offshore mixing is not an issue in such settings. Atmospheric evasion legacy resulting from wind events occurring several days prior to the actual survey is not likely in rivers either, because rivers are too dynamic systems in general. The legacy of storm events might be an issue in lakes, though. Hence, it is suggested to take consideration of the wind situation observed prior to radon survey activities in case of lake survey.

## Data Availability

The datasets generated during and/or analysed during the current study are available from the corresponding author on reasonable request.

## References

[CR1] Bender ML, Kinter S, Cassar N, Wanninkhof R (2011). Evaluating gas transfer velocity parameterizations using upper ocean radon distributions. Journal of Geophysical Research: Oceans.

[CR2] Black FJ, Paytan A, Knee KL, de Sieyes NR, Ganguli PM, Gray E, Flegal AR (2009). Submarine groundwater discharge of total mercury and monomethylmercury to central California coastal waters. Environmental Science & Technology.

[CR3] Burnett WC, Aggarwal PK, Aureli A, Bokuniewicz H, Cable JE, Charette MA, Kontar E, Krupa S, Kulkarni KM, Loveless A, Moore WS, Oberdorfer JA, Oliveira J, Ozyurt N, Povinec P, Privitera AMG, Rajar R, Ramessur RT, Scholten J, Stieglitz T, Taniguchi M, Turner JV (2006). Quantifying submarine groundwater discharge in the coastal zone via multiple methods. Science of the Total Environment.

[CR4] Burnett WC, Dulaiova H (2003). Estimating the dynamics of groundwater input into the coastal zone via continuous radon-222 measurements. Journal of Environmental Radioactivity.

[CR5] Burnett WC, Peterson R, Moore WS, de Oliveira J (2008). Radon and radium isotopes as tracers of submarine groundwater discharge—Results from the Ubatuba, Brazil SGD assessment intercomparison. Estuarine, Coastal and Shelf Science.

[CR6] Bussmann I, Suess E (1998). Groundwater seepage in Eckernförde Bay (Western Baltic Sea): Effect on methane and salinity distribution of the water column. Continental Shelf Research.

[CR7] Cable JE, Martin JB, Swarzenski PW, Lindenberg MK, Steward J (2004). Advection within shallow pore waters of a coastal lagoon, Florida. Ground Water.

[CR8] Chanyotha S, Kranrod C, Kritsananuwat R, Lane-Smith D, Burnett WC (2016). Optimizing laboratory-based radon flux measurements for sediments. Journal of Environmental Radioactivity.

[CR9] Cook PG, Rodellas V, Andrisoa A, Stieglitz TC (2018). Exchange across the sediment-water interface quantified from porewater radon profiles. Journal of Hydrology.

[CR10] Destouni, G., Hannerz, F., Prieto, C., Jarsjö, J., & Shibuo, Y. (2008). Small unmonitored near-coastal catchment areas yielding large mass loading to the sea. *Global Biogeochemical Cycles, 22*, GB4003.

[CR11] Dietze H, Löptien U (2021). Retracing hypoxia in Eckernförde Bight (Baltic Sea). Biogeosciences Discussions.

[CR12] Dulaiova H, Gonneea ME, Henderson PB, Charette MA (2008). Geochemical and physical sources of radon variation in a subterranean estuary—Implications for groundwater radon activities in submarine groundwater discharge studies. Marine Chemistry.

[CR13] Gilfedder BS, Frei S, Hofmann H, Cartwright I (2015). Groundwater discharge to wetlands driven by storm and flood events: Quantification using continuous Radon-222 and electrical conductivity measurements and dynamic mass-balance modelling. Geochimica Et Cosmochimica Acta.

[CR14] Hannerz, F., & Destouni, G. (2006). Spatial characterization of the Baltic Sea drainage basin and its unmonitored catchments. *AMBIO: A Journal of the Human Environment, 35*, 214–219. 10.1579/05-a-022r.1 16989505

[CR15] HELCOM (2018). Sources and pathways of nutrients to the Baltic Sea. *Baltic Sea Environment Proceedings No. 153*, Baltic Marine Environment Protection Commission, Katajanokanlaituri 6 B FI-00160 Helsinki, Finland.

[CR16] Hu C, Muller-Karges FE, Swarzenski PW (2006). Hurricanes, submarine groundwater discharge, and Florida´s red tides. Geophysical Research Letters.

[CR17] IAEA-TECDOC-1595 (2008). Nuclear and isotopic techniques for the characterization of submarine groundwater discharge in coastal zones. IAEA TECDOC Series, https://www.iaea.org/publications/7925/nuclear-and-isotopic-techniques-for-the-characterization-of-submarine-groundwater-discharge-in-coastal-zones

[CR18] Jensen JB, Kuijpers A, Bennike O, Laier T, Werner F (2002). New geological aspects for freshwater seepage and formation in Eckernförde Bay, western Baltic. Continental Shelf Research.

[CR19] Karstensen J, Liblik T, Fischer J, Bumke K, Krahmann G (2014). Summer upwelling at the Boknis Eck time-series station (1982 to 2012)—A combined glider and wind data analysis. Biogeosciences.

[CR20] Knee KL, Paytan A (2011). Submarine groundwater discharge: A source of nutrients, metals, and pollutants to the coastal ocean. TreaTise on Estuarine and Coastal Science.

[CR21] Krall L, Trezzi G, Garcia-Orellana J, Rodellas V, Mörth C-M, Andersson P (2017). Submarine groundwater discharge at Forsmark, Gulf of Bothnia, provided by Ra isotopes. Marine Chemistry.

[CR22] Kwon, E. Y., Kim, G., Primeau, F., Moore, W. S., Cho, H. -M., DeVries, T., Sarmiento, J. L., Charette, M. A., Cho, Y. -K., 2014. Global estimate of submarine groundwater discharge based on an observationally constrained radium isotope model. *Geophysical Research Letters*, 2014GL061574.

[CR23] Lee DR (1977). A device for measuring seepage flux in lakes and estuaries. Limnology Annd Oceanography.

[CR24] Lee YW, Kim G, Lim W-A, Hwang DW (2010). A relationship between submarine groundwater-borne nutrients traced by Ra isotopes and the intensity of dinoflagellate red-tides occurring in the southern sea of Korea. Limnology and Oceanography.

[CR25] Lehmann A, Myrberg K (2008). Upwelling in the Baltic Sea—A review. Journal of Marine Systems.

[CR26] Luo X, Jiao JJ (2016). Submarine groundwater discharge and nutrient loadings in Tolo Harbor, Hong Kong using multiple geotracer-based models, and their implications of red tide outbreaks. Water Research.

[CR27] MacIntyre, S., Wanninkhof, R., Chanton, J. P., 1995. Trace gas exchange across the air–water interface in freshwater and coastal marine environments. In: Biogenic trace gases: Measuring emissions from soil and water (eds. P. A. Matson and R. C. Hariss) Blackwell Science, 52–97.

[CR28] Myrberg K, Andrejev O (2003). Main upwelling regions in the Baltic Sea—A statistical analysis based on three-dimensional modelling. Boreal Environment Research.

[CR29] Pempkowiak J, Szymczycha B, Kotwicki L (2010). Submarine groundwater discharge (SGD) to the Baltic Sea. Rocznik Ochrona Środowiska.

[CR30] Petermann E, Knöller K, Rocha C, Scholten J, Stollberg R, Weiß H, Schubert M (2018). Coupling end-member mixing analysis and isotope mass balancing (222-Rn) for differentiation of fresh and recirculated submarine groundwater discharge (SGD) into Knysna Estuary, South Africa. Journal of Geophysical Research: Oceans.

[CR31] Piekarek-Jankowska H (1996). Hydrochemical effects of submarine groundwater discharge to the Puck Bay (Southern Baltic Sea, Poland). Geographia Polonica.

[CR32] Povinec PP, Burnett WC, Beck A, Bokuniewicz H, Charette M, Gonneea ME, Groening M, Ishitobi T, Kontar E, Kwong LW, L., Marie, D.E.P., Moore, W.S., Oberdorfer, J.A., Peterson, R., Ramessur, R., Rapaglia, J., Stieglitz, T., & Top, Z.  (2012). Isotopic, geophysical and biogeochemical investigation of submarine groundwater discharge: IAEA-UNESCO intercomparison exercise at Mauritius Island. Journal of Environmental Radioactivity.

[CR33] Purkl S, Eisenhauer A (2004). Determination of radium isotopes and 222Rn in a groundwater affected coastal area of the Baltic Sea and the underlying sub-sea floor aquifer. Marine Chemistry.

[CR34] Rahman MDM, Lee Y-G, Kim G, Lee K, Han S (2013). Significance of submarine groundwater discharge in the coastal fluxes of mercury in Hampyeong Bay, Yellow Sea. Chemosphere.

[CR35] Rocha C, Veiga-Pires C, Scholten J, Knoeller K, Gröcke DR, Carvalho L, Anibal J, Wilson J (2016). Assessing land–ocean connectivity via submarine groundwater discharge (SGD) in the Ria Formosa Lagoon (Portugal): Combining radon measurements and stable isotope hydrology. Hydrology and Earth System Sciences.

[CR36] Rocha C, Wilson J, Scholten J, Schubert M (2015). Retention and fate of groundwater-borne nitrogen in a coastal bay (Kinvara Bay, Western Ireland) during summer. Biogeochemistry.

[CR37] Rodellas V, Stieglitz TC, Tamborski J, Beek PV, Andrisoa A, Cook PG (2021). Conceptual uncertainties in groundwater and porewater fluxes estimated by radon and radium mass balances. LimnoLogy and Oceanography.

[CR38] Rodellas V, Garcia-Orellana J, Masqué P, Feldman M, Weinstein Y (2015). Submarine groundwater discharge as a major source of nutrients to the Mediterranean Sea. Proceedings of the National Academy of Sciences.

[CR39] Saderne V, Fietzek P, Herman PMJ (2013). Extreme Variations of pCO_2_ and pH in a macrophyte meadow of the Baltic Sea in summer: Evidence of the effect of photosynthesis and local upwelling. PLoS ONE.

[CR40] Santos IR, Burnett WC, Chanton J, Dimova N, Peterson RN (2009). Land or ocean?: Assessing the driving forces of submarine groundwater discharge at a coastal site in the Gulf of Mexico. Journal of Geophysical Research.

[CR41] Santos IR, Chen X, Lecher AL, Sawyer AH, Moosdorf N, Rodellas V, Tamborski J, Cho H-M, Dimova N, Sugimoto R, Bonaglia S, Li H, Hajati M-C, Li L (2021). Submarine groundwater discharge impacts on coastal nutrient biogeochemistry. Nature Reviews Earth & Environment.

[CR42] Schlüter M, Sauter EJ, Andersen CE, Dahlgaard H, Dando PR (2004). Spatial distribution and budget for submarine groundwater discharge in Eckernförde Bay (Western Baltic Sea). Limnology and Oceanography.

[CR43] Schmidt A, Schlueter M, Melles M, Schubert M (2008). Continuous and discrete on-site detection of radon-222 in ground- and surface waters by means of an extraction module. Applied Radiation and Isotopes.

[CR44] Schubert, M., Knoeller, K., Einsiedl F., & Rocha, C. (2015). Preliminary Evaluation of groundwater contributions to the water budget of Kinvarra Bay, Ireland, using ^222^Rn, EC and stable isotopes as natural indicators. *Environmental Monitoring and Assessment, 187*(3), 4274. 10.1007/s10661-015-4274-3 10.1007/s10661-015-4274-3 25666648

[CR45] Schubert M, Paschke A, Lieberman E, Burnett WC (2012). Air-Water Partitioning of ^222^Rn and its Dependence on Water Temperature and Salinity. Environmental Science & Technology.

[CR46] Schubert, M., Petermann, E., Stollberg, R., Gebel, M., Scholten, J., Knöller, K., Lorz, C., Glück, F., Riemann, K., & Weiss, H. (2019). Improved Approach for the Investigation of Submarine Groundwater Discharge by Means of Radon Mapping and Radon Mass Balancing. *Water, 11*, 749. 10.3390/w11040749

[CR47] Schubert M, Scholten J, Schmidt A, Comanducci J, Pham M, Mallast U, Knoeller K (2014). Submarine groundwater discharge at a single spot location: Evaluation of different detection approaches. Water.

[CR48] Seitzinger, S. P., & Harrison, J. A. (2008). Land-based N sources and their delivery to coastal systems, in: D. Capone, D.A. Bronk, M.R. Mullholland, E. Carpenter (Eds.), Nitrogen in the marine environment, 2nd edition. Academic Press, New York.

[CR49] Stieglitz TC, Cook PG, Burnett WC (2010). Inferring coastal processes from regional-scale mapping of 222Radon and salinity: Examples from the Great Barrier Reef, Australia. Journal of Environmental Radioactivity.

[CR50] Taniguchi M, Burnett WC, Cable JE, Turner JV, Wang K, Gamo T, Wang K, Gamo T (2003). Assessment methodologies for submarine groundwater discharge.

[CR51] Trezzi G, Garcia-Orellana J, Santos-Echeandia J, Rodellas V, Garcia-Solsona E, Garcia-Fernandez, & G., Masqué, P.  (2016). The influence of a metal-enriched mining waste deposit on submarine groundwater discharge to the coastal sea. Marine Chemistry.

[CR52] Vanek V, Lee DR (1991). Mapping submarine groundwater discharge areas—An example from Laholm Bay, Southwest Sweden. Limnology and Oceanography.

[CR53] von Ahn CME, Scholten JC, Malik C, Feldens P, Liu B, Dellwig O, Jenner A-K, Papenmeier S, Schmiedinger I, Zeller MA, Böttcher ME (2021). A multi-tracer study of fresh water sources for a temperate urbanized coastal bay (Southern Baltic Sea). Frontiers in Environmental Science.

